# Distinct Microbial Community of Accumulated Biofilm in Dental Unit Waterlines of Different Specialties

**DOI:** 10.3389/fcimb.2021.670211

**Published:** 2021-06-17

**Authors:** Cancan Fan, Haijing Gu, Limin Liu, Haiwei Zhu, Juan Yan, Yongbiao Huo

**Affiliations:** ^1^ Zhujiang New Town Clinic, Hospital of Stomatology, Guanghua School of Stomatology, Sun Yat-sen University, Guangzhou, China; ^2^ Guangdong Provincial Key Laboratory of Stomatology, Guangzhou, China

**Keywords:** dental unit waterlines, microbial community, biofilm, pathogens, high-throughput sequencing

## Abstract

The contamination of dental unit waterlines (DUWLs) is a serious problem and directly affects the dental care. This study aims to explore the microbial community of biofilm in DUWL from different specialties and investigate the associated factors. A total of 36 biofilm samples from 18 DUWL of six specialties (*i.e.*, prosthodontics, orthodontics, pediatrics, endodontics, oral surgery, and periodontics) at two time points (*i.e.*, before and after daily dental practice) were collected with a novel method. Genomic DNA of samples was extracted, and then 16S ribosomal DNA (rDNA) (V3–V4 regions) and ITS2 gene were amplified and sequenced. Kruskal–Wallis and Wilcoxon rank test were adopted for statistical analysis. Microbial community with high diversity of bacteria (631 genera), fungi (193 genera), and viridiplantae was detected in the biofilm samples. Proteobacteria was the dominant bacteria (representing over 65.74–95.98% of the total sequences), and the dominant fungi was Ascomycota (93.9–99.3%). Microorganisms belonging to multiple genera involved in human diseases were detected including 25 genera of bacteria and eight genera of fungi, with relative abundance of six genera over 1% (*i.e.*, *Acinetobacter*, *Pseudomonas*, *Enterobacter*, *Aspergillus*, *Candida*, and *Penicillium*). The biofilm microbiome may be influenced by the characteristics of dental specialty and routine work to some extent. The age of dental chair unit and overall number of patients had the strongest impact on the overall bacteria composition, and the effect of daily dental practices (associated with number of patients and dental specialty) on the fungi composition was the greatest. For the first time, biofilm in DUWL related to dental specialty was comprehensively evaluated, with more abundance of bacterial and fungal communities than in water samples. Biofilm accumulation with daily work and multiple kinds of opportunistic pathogen emphasized the infectious risk with dental care and the importance of biofilm control.

## Introduction

Dental unit waterlines (DUWLs) include air/water syringe, ultrasonic scaler, and narrow-bore plastic tubing that carry water to the dental instruments. Contamination is often observed in the complex DUWL with high densities of microorganisms, such as bacteria, fungi, viruses, and protozoa (Fujita et al., 2017; Spagnolo et al., 2019). The contamination may be caused by the water supply (Zhang et al., 2018), the retraction of biological fluids from the handpieces used in oral cavities of patients (Costa et al., 2015), or probably the continuous biofilm detachment or fragmentation in the narrow waterline tubes (Lal et al., 2017).

Except for some harmless microorganisms (*e.g.*, *Flavobacterium* and *Moraxella*) (Lizzadro et al., 2019), opportunistic pathogens such as *Legionella pneumophila*, *Pseudomonas aeruginosa*, *Mycobacterium*, *Staphylococcus aureus* and amoebae have previously been revealed in water samples from DUWL (Güngör et al., 2014; Spagnolo et al., 2019). In addition, other genera such as *Propionibacterium* and *Stenotrophomonas* were also recovered in dental unit waters (Fotedar and Ganju, 2015). Several reports have informed diseases associated with DUWL, especially pneumonia caused by *Legionella pneumophila* (Ricci et al., 2012; Schönning et al., 2017). Facial cutaneous sinus tract associated with *Mycobacterium fortuitum*, *M. abscessus*, and *M. peregrinum* in the DUWL were also reported recently (Perez-Alfonzo et al., 2020). In fact, both patients and dental staff are regularly exposed to multifarious infectious risks due to inhalation or spreading of aerosols produced during dental cares. However, infections could be underestimated because the associations between infection and recent exposure to contaminated dental water or aerosols were difficult to confirm (Arvand and Hack, 2013), on the other hand, the true condition of contamination in DUWL was ambiguous.

Investigation of the bacterial communities present in DUWL was once performed with the help of pyrosequencing (Costa et al., 2015), and it verified that a high bacterial and fungal diversity remained in the output water despite disinfecting treatment and flushing process (Costa et al., 2016a). (Ji et al., 2018) summarized three key factors of influence as follows: daily or weekly disinfection of DUWL, water supply source, and dental chair unit (DCU) with a valid anti-retraction valve. As we know, the narrow-bore tubing encourages biofilm formation with a very large ratio of surface area to volume (6:1) (Walker and Marsh, 2007). Research showed that silver coating applied to the luminal surface of the commercial waterline tubing failed to prevent biofilm formation (Lal et al., 2017). Biofilms up to 50-μm thick have been found in functioning dental units, composed of complex microbial communities (Porteous et al., 2011). Yeasts once identified in DUWL especially *Candida* can form biofilms and resist antifungal agents (Mazari et al., 2018). As a result, no matter how various procedures were applied, none of these can stop biofilm from accumulating and detaching. On the basis of comprehensive research on biofilms, further studies on prevention of biofilm accumulation are essential. It is pivotal to monitor and assess a full pattern of microbial contamination in DUWL of different specialties.

Methods of culturing organisms from DUWL samples (the circulation of water within the DUWL and turbine handpiece output water) and providing the heterotrophic plate count [HPC, calculated as colony forming units (CFUs)/mL] of living bacteria in water samples were applied in most of the previous research studies about DUWL. However, these methods may fail to detect all microorganisms (including those uncultivable microbes) and the actual diversity in DUWL. The aim of this study was to investigate both bacterial and fungal communities which were two of the major contaminants in the biofilm samples of DUWL, using high-throughput sequencing technology. With the purpose of systematically understanding the microbial communities and revealing the associations with environmental factors, the age of DCU, overall number of patients, and dental practices of different specialties together with different sampling time points were incorporated into this study. Our findings could help better characterize and assess the cross-contamination risk of dental care and suggest the information of better biofilm control with more pertinence in DUWL.

## Materials and Methods

### Dental Unit Biofilm Sample Collection

Eighteen dental units used daily for dental care were chosen from one dental clinic, which were for specialties of prosthodontics, orthodontics, pediatrics, endodontics, oral surgery, and periodontics. The water supply of turbine handpiece was purified water offered by the same company. The disinfection system was chlorinated disinfectant (weekly used) and was replaced with a filter unit (Dentapure, Crosstex, Chinese) in succession lately, which was supervised by SenSafe (lodine check). Operating years, overall number of patients per year (replaced with average patients treated per month) together with the number of patients on the sampling day of DCU from different specialties were shown in **Table 1**. Flushing the DUWL for 30 s and sterilization of handpieces were all performed before the first patient and after each patient.

**Table 1:** The general information of sampling Dental Chair Units.

	Specialty	Sample label	Application of filter unit	Age	Average patients per month	Number of patients on the sampling day
Group1	Prosthodontic	1	5 months	<1 year	210+	7
		2	5 months	<1 year	230+	5
		3	5 months	<1 year	180+	3
		10	5 months	<1 year	240+	13
		14	5 months	8–10 years	240+	7
Group2	Orthodontics	4	1.5 month	8–10 years	340+	12
		5	1.5 month	3–5 years	260+	13
		6	1.5 month	8–10 years	260+	10
Group3	Pediatrics	7	1.5 month	8–10 years	310+	12
		8	1.5 month	3–5 years	730+	14
		9	1.5 month	3–5 years	340+	9
Group4	Endodontics	11	5 months	8–10 years	140+	13
		12	5 months	8–10 years	230+	11
		16	1.5 month	<1 year	300+	16
Group5	Oral surgery	13	1.5 month	<1 year	200+	8
		18	1.5 month	8–10 years	270+	6
Group6	Periodontics	15	1.5 month	<1 year	280+	8
		17	1.5 month	<1 year	330+	9

DUWL samples were collected from the plastic tube concatenated to high-speed handpiece using sterilized mini brush by the same sampler. The brush was slender enough (brush of kernel: diameter 3 mm, length 3.8 cm; total length: 17 cm) to insert into the tube, then rotated and traversed for 10 times to collect enough biofilm attached to the tube wall. Brush of kernel was broken with sterilized plier and preserved in EP tube. The tubes were immediately transported on ice to the laboratory within half an hour and restored at −80°C no longer than one month before further process.

During sampling, protective measures including sterile gloves and facial mask were adopted to eliminate microorganism’s contamination from the sampler. The two sampling time periods were 7:30–8:00 in the morning before the routine work (group M) and 17:00–17:30 in the afternoon when clinical work was finished on the same day before daily disinfection (group N). Information including age of DCU, disinfection system, and the application of filter unit, overall number of patients in recent year of each DCU, number of patients, and specialty of dental practice on the sampling day was recorded. The dentists of different specialties were usually fixed with the same DCU.

### DNA Extraction and PCR Amplification

The mini brush with biofilm samples were oscillated with ddH_2_O, then centrifuged at 8,000 g for 3 min. The sediment was collected and suspended in 500 μl of ddH_2_O. Genomic DNA was extracted using the HiPure Stool DNA Kits (Magen, Guangzhou, China) according to the manufacturer’s protocols. Primers (341F: CCTACGGGNGGCWGCAG; 806R: GGACTACHVGGGTATCTAAT) were used for the bacterial DNA amplification (the V3–V4 hypervariable regions of the bacterial 16S rDNA gene) (Guo et al., 2017). Primers (ITS3_KYO2: GATGAAGAACGYAGYRAA; ITS4: TCCTCCGCTTATTGATATGC) were used for the fungal ITS2 gene amplification (Toju et al., 2012). According to the same manufacturer’s protocol (Toyobo, Osaka, Japan), PCR reactions were performed in triplicate 50 μl mixture containing 5 μl of 10× KOD Buffer, 5 μl of 2 mM dNTPs, 3 μl of 25 mM MgSO_4_, 1.5 μl of each primer (10 μM), 1 μl of KOD Polymerase, and 100 ng of template DNA (94°C for 2 min, followed by 30 cycles at 98°C for 10 s, 62°C for 30 s, and 68°C for 30 s and a final extension at 68°C for 5 min). Then, the products of PCR amplification were collected by gel cutting and quantified using ABI StepOnePlus Real-Time PCR System (Life Technologies, Foster City, USA).

### Sequencing of 16S and ITS Gene

The purified amplification products were pooled in equimolar and paired-end sequenced (2 × 250) on an Illumina platform (Hiseq2500 PE250) following the manufacturer’s recommendations. Noisy sequences of raw tags were filtered by QIIME (Caporaso et al., 2010) (version 1.9.1) to obtain the high-quality clean tags. The effective tags were clustered into operational taxonomic units (OTUs) of ≥ 97% similarity using UPARSE (version 9.2.64) pipeline (Edgar, 2013). The raw reads have been deposited into the NCBI Sequence Read Archive (SRA) database (Accession Number: PRJNA664509).

### Statistical Analysis

The microbial community was analyzed in terms of descriptive statistics. Alpha-diversity (the Chao1 richness, ACE indices, Simpson and Shannon diversity indices) and Beta-diversity [Jaccard, Bray–Curtis, principal coordinates analysis (PCoA), non-metric multi-dimensional scaling analysis (NMDS)] was calculated with QIIME. Samples were classified into six groups according to the characteristics of dental practice: group 1—prosthodontics, group 2—orthodontics, group 3—pediatrics, group 4—endodontics, group 5—oral surgery, group 6—periodontics. The differences among the six groups of specialty (groups 1–6) and between samples in the morning and afternoon (groups M, N) were evaluated by means of a Kruskal–Wallis test and a non-parametric Wilcoxon rank-sum test in R project Vegan package (version 2.5.3). Multivariate statistical techniques including PCoA and NMDS of Jaccard and Bray–Curtis distances were also generated. Indicator species analysis was performed using Welch’s t-test and Wilcoxon rank test. The associations between microbiota composition and environmental factors (age of DCU, time of filter unit application, overall number of patients, number of patients on the sampling day, and specialty of dental practice) were evaluated with Pearson correlation analysis. Heatmap and network of correlation coefficient were generated using Omicsmart, a dynamic real-time interactive online platform for data analysis (http://www.omicsmart.com). A *p* value <0.05 was considered statistically significant.

## Results

### Microbial Community Composition of Overall DUWL Biofilm Samples

**Figure 1:**
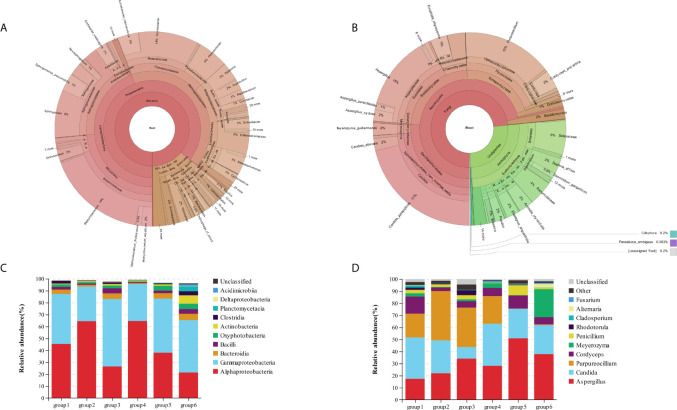
The relative abundance of bacterial **(A)** and fungal **(B)** genera in the DUWL core microbiome; relative abundance of bacterial community compositions at class level in six groups **(C)**; relative abundance of fungal community compositions at genus level in six groups **(D)**.

In our study, microorganisms including bacteria, fungi, viridiplantae, and protists were clearly detected in the biofilm samples of DUWL. A total of 1,762,370 bacterial reads (mean length: 456 bases) and 543,175 fungal reads (mean length: 361 bases) were obtained from the 36 dental unit biofilm samples indicating a high microbial diversity in DUWL. The 16S rDNA gene sequencing showed that the bacterial communities of all samples covered 31 phyla, 86 classes, 177 orders, 306 families, 631 genera, and 350 species. The ITS2 gene sequencing showed that the fungal communities covered four phyla, 21 classes, 59 orders, 130 families, 193 genera, and 169 species. The relative abundance of bacterial and fungal genera in the DUWL core microbiome and the top ten classes of bacteria and genera of fungi were illustrated in **Figure 1**.

The overall relative abundances (%) of the top six bacteria and fungi at phylum, class, order, family, and genus level and that among six groups of DUWL biofilm samples before/after daily dental practice were shown in **Table 2**. At phylum level, Proteobacteria was the most dominant bacteria (representing over 65.7–96.0% of the total sequences) and that of fungi was Ascomycota (93.9–99.3%) in all samples. Other phyla of bacteria including Firmicutes, Bacteroidetes, Cyanobacteria, Actinobacteria, Planctomycetes, Acidobacteria, as well as the fungi of Basidiomycota were also found in biofilm samples. Among the six groups, Alphaproteobacteria and Gammaproteobacteria were the same dominant classes of bacteria; *Methylobacterium*, *Acinetobacter*, and *Sphingobium* were the most abundant genera. The dominant genera of fungi among six groups were as follows respectively: groups 1/2/4 were *Aspergillus*, *Candida*, and *Purpureocillium*; group 3 were *Aspergillus*, and *Purpureocillium*; group 5/6 were *Aspergillus* and *Candida*. Based on the information above, *Aspergillus* was the dominant genus of fungi among all groups. *Candida_parapsilosis*, *Cordyceps_polyarthra*, and *Meyerozyma_guilliermondii* were the most abundant fungal species.

**Table 2:** The overall relative abundance (%) of top six bacteria and fungi at phylum, class, order, family and genus levels and that among six groups of DUWL biofilm samples at two time points (before/after daily dental practice) (relative abundance >0.1%).

		Group 1	Group 2	Group 3	Group 4	Group 5	Group 6	Overall
		Before/After Daily Dental Practice						
**Bacteria**								
**Phylum**	Proteobacteria	85.0/90.0	93.5/93.5	87.3/79.8	98.3/93.7	85.8/81.8	64.6/66.9	86.4
	Firmicutes	6.6/2.6	1.8/1.5	4.6/7.1	0.3/1.7	2.1/3.8	13.5/2.0	3.9
	Bacteroidetes	4.3/2.9	1.5/1.4	2.8/6.7	0.3/1.1	4.4/4.7	6.2/4.6	3.3
	Cyanobacteria	2.4/2.3	0.9/1.4	2.3/2.2	0.2/1.2	3.5/4.6	2.2/6.9	2.3
	Actinobacteria	0.8/1.1	1.1/1.0	1.4/1.8	0.4/0.7	1.2/1.6	8.2/9.4	1.9
	Planctomycetes	0.1/0.2	0.4/0.1	0.1/0.1	0.2/0.1	0.5/1.5	0.1/8.5	0.7
**Class**	Alphaproteobacteria	44.9/46.0	67.5/61.5	26.1/26.8	70.1/59.2	37.6/38.5	31.4/11.4	45.1
	Gammaproteobacteria	40.0/44.0	26.0/31.9	61.1/52.1	28.2/33.9	48.0/42.8	32.6/55.2	41.0
	Bacteroidia	4.3/2.9	1.5/1.4	2.8/6.7	0.3/1.1	4.4/4.7	6.2/4.6	3.3
	Bacilli	2.6/1.8	1.3/1.1	3.9/4.7	0.2/1.3	1.7/2.5	6.6/1.1	2.3
	Oxyphotobacteria	1.9/2.2	0.9/1.4	2.2/2.2	0.2/1.1	3.5/4.5	2.2/6.8	2.2
	Actinobacteria	0.7/0.9	0.8/1.0	1.2/1.4	0.4/0.6	1.1/1.6	6.9/7.0	1.6
**Order**	Rhizobiales	16.0/24.0	55.6/51.6	8.6/8.9	30.0/24.1	28.6/30.2	15.8/4.2	24.9
	Pseudomonadales	24.3/24.0	7.5/15.9	30.7/27.9	6.1/14.2	29.9/24.6	12.5/4.0	19.2
	Sphingomonadales	26.9/20.3	11.1/7.2	6.6/5.8	38.8/33.4	7.2/6.7	8.0/2.0	16.5
	Betaproteobacteriales	6.4/7.5	11.3/9.1	7.9/5.9	17.8/13.4	12.2/11.0	9.6/9.9	9.7
	Enterobacteriales	7.7/10.1	5.2/6.4	8.6/7.9	3.7/5.0	2.5/2.3	6.0/1.5	6.2
	Xanthomonadales	1.2/1.9	0.5/0.4	12.5/10.1	0.3/0.9	2.1/3.3	3.3/5.3	3.3
**Family**	Beijerinckiaceae	9.5/19.5	53.0/46.5	3.9/4.6	29.4/22.1	24.5/24.2	14.6/1.8	20.9
	Sphingomonadaceae	26.9/20.3	11.1/7.2	6.6/5.8	38.8/33.4	7.2/6.8	8.0/2.0	16.5
	Moraxellaceae	18.8/16.2	5.5/13.1	21.9/19.2	0.7/9.3	27.1/20.7	10.4/2.5	14.1
	Burkholderiaceae	5.9/7.2	11.1/8.9	7.7/5.7	12.2/9.4	11.9/11.0	9.3/8.1	8.6
	Enterobacteriaceae	7.7/10.1	5.2/6.4	8.6/7.9	3.7/5.0	2.5/2.2	6.0/1.5	6.2
	Pseudomonadaceae	5.5/7.7	2.0/2.7	8.8/8.7	5.4/5.0	2.8/3.9	2.1/1.5	5.1
**Genus**	*Methylobacterium*	9.3/19.3	53.0/45.9	3.8/4.5	28.7/21.4	24.3/23.9	13.9/1.4	20.6
	*Acinetobacter*	18.2/15.6	5.2/12.6	21.3/18.7	0.6/9.1	26.6/20.3	10.0/2.1	13.6
	*Sphingobium*	14.3/9.6	2.3/0.3	0.14/0.32	22.8/21.0	1.9/1.6	2.0/0.7	7.6
	*Sphingomonas*	5.5/5.8	6.4/3.3	4.1/3.3	9.7/8.7	4.0/3.6	3.8/0.9	5.2
	*Pseudomonas*	5.5/7.7	2.0/2.7	8.8/8.7	5.4/5.0	2.8/3.9	2.1/1.5	5.1
	*Stenotrophomonas*	1.2/1.9	0.5/0.4	12.2/10.1	0.2/0.6	2.0/3.2	2.9/5.3	3.2
**Fungi**								
**Phylum**	Ascomycota	98.3/94.5	98.9/99.0	95.5/92.4	99.5/99.2	98.7/98.6	99.6/98.7	97.4
	Basidiomycota	1.7/5.4	1.1/0.8	4.3/7.6	0.25/0.79	1.2/1.4	0.4/1.3	2.5
**Class**	Sordariomycetes	35.7/34.5	44.3/44.7	35.6/43.0	38.0/24.2	15.8/8.8	9.2/6.8	31.8
	Saccharomycetes	34.5/40.1	31.1/25.6	13.8/9.4	40.4/36.8	25.2/24.8	56.6/29.1	31.0
	Eurotiomycetes	23.5/17.4	22.6/26.1	44.0/37.1	20.8/36.8	55.8/63.7	28.2/60.4	32.1
	Dothideomycetes	4.3/2.1	0.8/1.7	1.5/1.0	0.3/1.3	1.5/1.0	5.3/0.8	1.9
	Microbotryomycetes	0.9/3.2	1.0/0.6	3.4/4.6	0.0/0.0	0.4/0.6	0.0/0.0	1.5
	Agaricomycetes	0.6/0.6	0.0/0.1	0.4/2.2	0.2/0.7	0.2/0.3	0.1/0.2	0.5
**Order**	Hypocreales	34.4/34.3	44.0/44.3	35.1/41.5	37.9/23.7	15.3/7.5	8.0/6.4	31.2
	Saccharomycetales	34.5/40.1	31.1/25.6	13.8/9.4	40.4/36.8	25.2/24.8	56.6/29.1	31.0
	Eurotiales	23.3/17.2	22.5/26.0	41.1/34.9	20.8/36.8	55.8/63.7	28.2/60.4	31.6
	Sporidiobolales	0.9/3.2	1.0/0.6	3.4/4.6	0.0/0.0	0.4/0.6	0.0/0.0	1.5
	Pleosporales	1.3/1.4	0.7/0.1	0.8/0.5	0.1/0.1	1.0/0.2	4.8/0.7	0.9
	Capnodiales	2.9/0.6	0.0/0.5	0.6/0.4	0.2/1.2	0.5/0.8	0.6/0.1	0.8
**Family**	Aspergillaceae	23.2/17.2	22.5/26.0	40.8/34.4	20.8/36.8	55.3/63.6	28.2/60.4	31.5
	Saccharomycetales_fam_Incertae_sedis	33.0/35.7	30.8/23.7	12.2/6.9	36.0/33.6	24.9/24.2	21.5/29.0	27.0
	Ophiocordycipitaceae	20.1/19.3	41.4/40.3	31.1/34.2	24.2/21.6	0.1/0.4	0.8/0.1	22.2
	Cordycipitaceae	14.1/14.4	2.5/3.6	3.7/7.0	12.1/1.7	15.0/7.0	5.8/6.3	8.4
	Debaryomycetaceae	1.4/4.3	0.1/0.7	1.1/2.3	4.5/3.2	0.2/0.4	34.9/0.1	3.8
	Sporidiobolaceae	0.9/3.2	1.0/0.6	3.4/4.6	0.0/0.0	0.4/0.6	0.0/0.0	1.5
**Genus**	*Candida*	33.0/35.7	30.8/23.8	12.2/6.9	35.9/33.6	24.9/24.0	21.5/29.0	26.9
	*Aspergillus*	20.0/14.6	19.8/24.8	38.0/30.3	20.0/36.4	48.5/53.4	27.1/59.3	28.5
	*Purpureocillium*	20.1/19.3	41.4/40.3	31.0/34.2	24.2/21.6	0.0/0.0	0.8/0.1	22.2
	*Cordyceps*	14.0/14.3	2.4/3.6	3.7/7.0	12.0/1.7	14.9/6.7	5.8/6.3	8.4
	*Meyerozyma*	1.4/4.3	0.1/0.7	1.0/2.3	4.5/3.2	0.2/0.4	34.9/0.1	3.8
	*Penicillium*	3.1/2.3	2.7/1.9	2.8/4.1	0.6/0.3	6.7/9.9	0.7/1.1	2.8

Group 1—prosthodontics; group 2—orthodontics; group 3—pediatrics; group 4—endodontics; group 5—oral surgery; group 6—periodontics.

Certain amount of potential human pathogens was detected in the biofilm samples, including 25 genera of bacteria and eight genera of fungi. The overall relative abundances (%) of potential pathogenic microorganism at genus level and that among six groups of DUWL biofilm samples were shown in **Table 3**. The most abundant six genera with relative abundance over 1% were *Acinetobacter*, *Pseudomonas*, *Enterobacter*, *Aspergillus*, *Candida*, and *Penicillium*. Some reported representative opportunistic pathogens associated with DUWL like *Methylophilus*, *Escherichia-Shigella*, *Legionella*, *Streptococcus*, and *Flavobacterium* were all detected. In this study, we also detected opportunistic pathogens including bacteria of *Acinetobacter_lwoffii*, *Actinomyces_gerencseriae, Afipia_genosp, Chlamydia_trachomatis, Clostridium_perfringens, Edwardsiella_tarda, Endobacter_medicaginis, Gardnerella_vaginalis, Neisseria_gonorrhoeae, Serratia_marcescens, Shinella_zoogloeoides, Staphylococcus_aureus, Streptococcus_pneumoniae, Streptococcus* spp, *Prevotella* spp, and fungi of *Candida_albicans, Exophiala_dermatitidis, Fusarium_solani, Stachybotrys_chartarum, Trichothecium_roseum, Trichoderma_spirale.* Among these established microorganisms, almost all of the opportunistic pathogens were distributed in the six groups of samples, except that *Methylophilus* was not detected in group 1 (prosthodontics), *Haemophilus* was not detected in group 5 (oral surgery), *Klebsiella* and *Stachybotrys* were not detected in groups 5 (oral surgery) and 6 (periodontics). Moreover, *Gardnerella* could be only detected in group 4 (endodontics), while *Exophiala* was only detected in groups 1 (prosthodontics), 2 (orthodontics), and 3 (pediatrics).

**Table 3:** The overall relative abundance (%) of potential pathogenic microorganism at genus level and that among six groups of DUWL biofilm samples.

Pathogen	Group1	Group2	Group3	Group4	Group5	Group6	Overall
**Bacteria**							
*Acinetobacter*	16.924	8.858	20.019	4.856	23.445	6.069	13.362
*Pseudomonas*	6.610	2.370	8.760	5.200	3.360	1.835	4.689
*Enterobacter*	2.925	1.625	2.682	0.489	0.395	0.765	1.480
*Bacillus*	1.005	0.318	1.066	0.309	0.616	0.837	0.692
*Staphylococcus*	0.138	0.034	0.157	0.132	0.962	2.009	0.572
*Nocardioides*	0.131	0.015	0.035	0.015	0.020	0.804	0.170
*Edwardsiella*	0.294	0.225	0.199	0.021	0.165	0.002	0.151
*Bacteroides*	0.452	0.133	0.062	0.021	0.013	0.217	0.150
*Neisseria*	0.192	0.066	0.076	0.004	0.036	0.359	0.122
*Serratia*	0.045	0.069	0.050	0.006	0.109	0.365	0.107
*Streptococcus*	0.182	0.061	0.141	0.022	0.084	0.136	0.104
*Actinomyces*	0.099	0.178	0.041	0.001	0.117	0.080	0.086
*Flavobacterium*	0.061	0.011	0.045	0.056	0.052	0.231	0.076
*Escherichia-Shigella*	0.153	0.058	0.049	0.008	0.053	0.041	0.060
*Mycobacterium*	0.014	0.035	0.043	0.036	0.022	0.199	0.058
*Aeromonas*	0.021	0.035	0.197	0.005	0.05	0.001	0.052
*Rhodococcus*	0.027	0.005	0.089	0.007	0.031	0.135	0.049
*Vibrio*	0.024	0.012	0.017	0.007	0.042	0.186	0.048
*Prevotella_7*	0.030	0.032	0.001	0.003	0.01	0.181	0.043
*Klebsiella*	0.005	0.197	0.005	0.001	0	0	0.035
*Legionella*	0.028	0.006	0.074	0.002	0.039	0.014	0.027
*Corynebacterium*	0.019	0.010	0.001	0.006	0.034	0.043	0.019
*Haemophilus*	0.018	0.015	0.033	0.008	0	0.011	0.014
*Methylophilus*	0	0.002	0.004	0.002	0.016	0.039	0.011
*Gardnerella*	0	0	0	0.036	0	0	0.006
**Fungi**							
*Aspergillus*	17.344	21.918	34.133	28.222	50.971	37.855	28.518
*Candida*	34.321	27.286	9.574	34.760	24.453	24.020	26.921
*Penicillium*	2.696	2.289	3.402	0.451	8.284	0.857	2.848
*Cladosporium*	1.662	0.254	0.458	0.610	0.620	0.290	0.791
*Alternaria*	0.363	0.014	0.031	0.068	0.178	3.231	0.410
*Fusarium*	0.054	0.003	0.050	0.710	0.114	0.750	0.220
*Exophiala*	0.005	0.069	0.525	0	0	0	0.103
*Stachybotrys*	0.023	0.054	0.013	0.007	0	0	0.019

### Microbial Community Diversity of Samples From Different Specialties

The complexity of the bacterial and fungal communities in the six groups of specialty from the DUWL biofilm samples was investigated based on richness and evenness, measured by alpha-diversity (ACE, Chao1, Shannon, Simpson and Goods_ coverage indices). The calculated Alpha-diversity indices are presented in **Table 4**. The similarities of groups were analyzed *via* Bray–Curtis calculation.

**Table 4:** Number of genus, alpha-diversity index and Good_coverage for bacteria of six groups.

	Specialty	Number of genus	ACE	Chao1	Shannon	Simpson	Good coverage
Group1	Prosthodontic	238	3,417	3,362	6.69	0.961	0.988
Group2	Orthodontics	207	2,763	2,716	6.27	0.963	0.990
Group3	Pediatrics	221	3,414	3,314	6.77	0.971	0.988
Group4	Endodontic	213	2,979	2,879	5.67	0.932	0.989
Group5	Oral surgery	196	3,491	3,318	6.91	0.973	0.989
Group6	Periodontics	208	3,014	2,778	6.58	0.935	0.991

**Figure 2:**
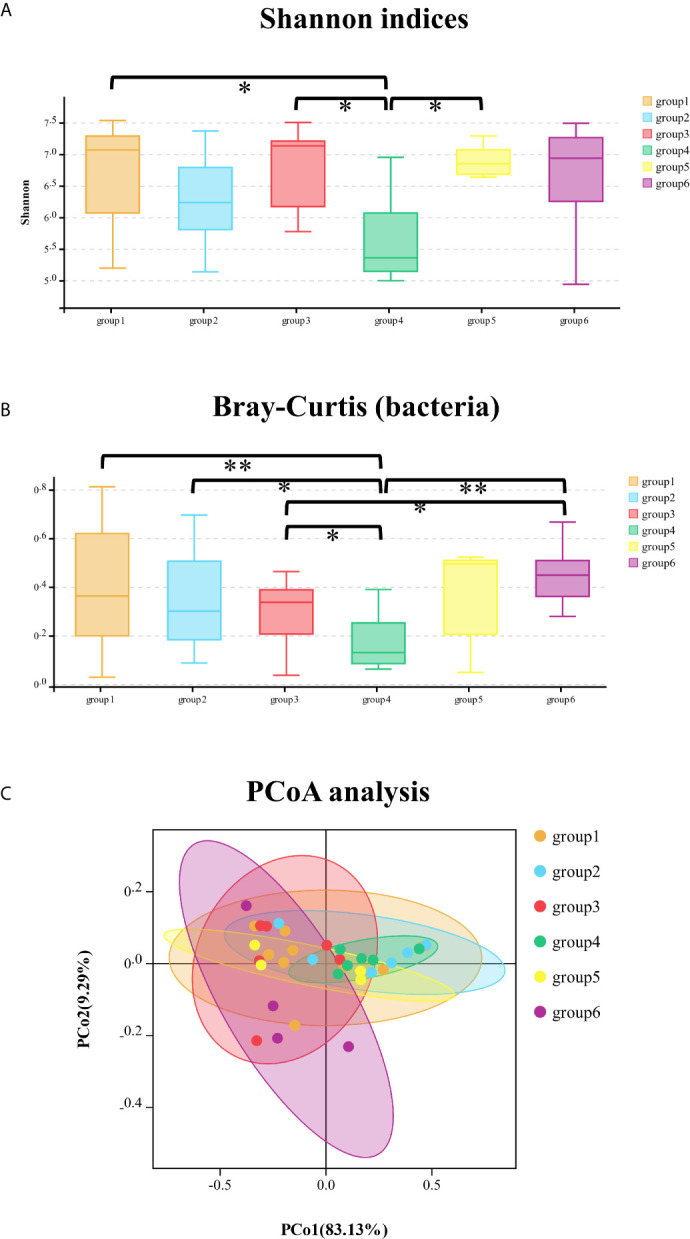
The comparison of Shannon indices of bacterial community among six groups at phylum level **(A)**; the comparison of Bray–Curtis distance of bacterial community among six groups at class level **(B)**; principal co-ordinates analysis (PCoA) of bacterial community among the six groups of samples **(C)**. *significant at *p* < 0.05, **significant at *p* < 0.01.

In the bacterial community, Shannon and Simpson indices were significantly different between groups 1/3/5 and group 4, which meant that the bacteria richness and evenness were different between the specialty of prosthodontics/pediatrics/oral surgery and endodontics (**Figure 2A** **)**. The specific different bacteria were as follows respectively: Proteobacteria (mean abundance: 87.52 *vs* 95.98%, *p* = 0.01), Firmicutes (4.60 *vs* 0.97%, *p* = 0.01), Bacteroidetes (3.61 *vs* 0.73%, *p* = 0.02), Cyanobacteria (2.36 *vs* 0.72%, *p* = 0.02) in group 1 and group 4; *Methylobacterium* (4.20 *vs* 25.02%, *p* = 0.02), *Sphingobium* (0.23 *vs* 21.90%, *p* = 0.03), *Ralstonia* (4.06 *vs* 1.39%, *p* = 0.01) in group 3 and group 4; *Sphingobium* (21.90 *vs* 1.72%, *p* = 0.04), *Ralstonia* (1.39 *vs* 3.13%, *p* = 0.04) in group 4 and group 5. The relative abundance of *Sphingobium* was significantly higher in group 4 (endodontics) than in other groups. According to the beta-diversity distance matrix, community structure of the six groups was significantly different with the calculation of Bray–Curtis and analysis at phylum (Kruskal–Wallis test, *p <* 0.001) and class level (Kruskal–Wallis test, *p* = 0.02). There’s an obvious separation of the bacterial communities between groups of samples at class level: groups 1/2/3/6 and group 4 (Wilcoxon test, *p <* 0.001/*p* = 0.01/*p* = 0.01/*p* = 0.002, respectively); group 3 and group 6 (Wilcoxon test, *p* = 0.04) (**Figure 2B**). Based on the weighted PCoA analysis, the first (PCO1) and second (PCO2) axes showed values of cumulative percentage variance of species equal to 78.07 and 14.10% (**Figure 2C**).

**Figure 3:**
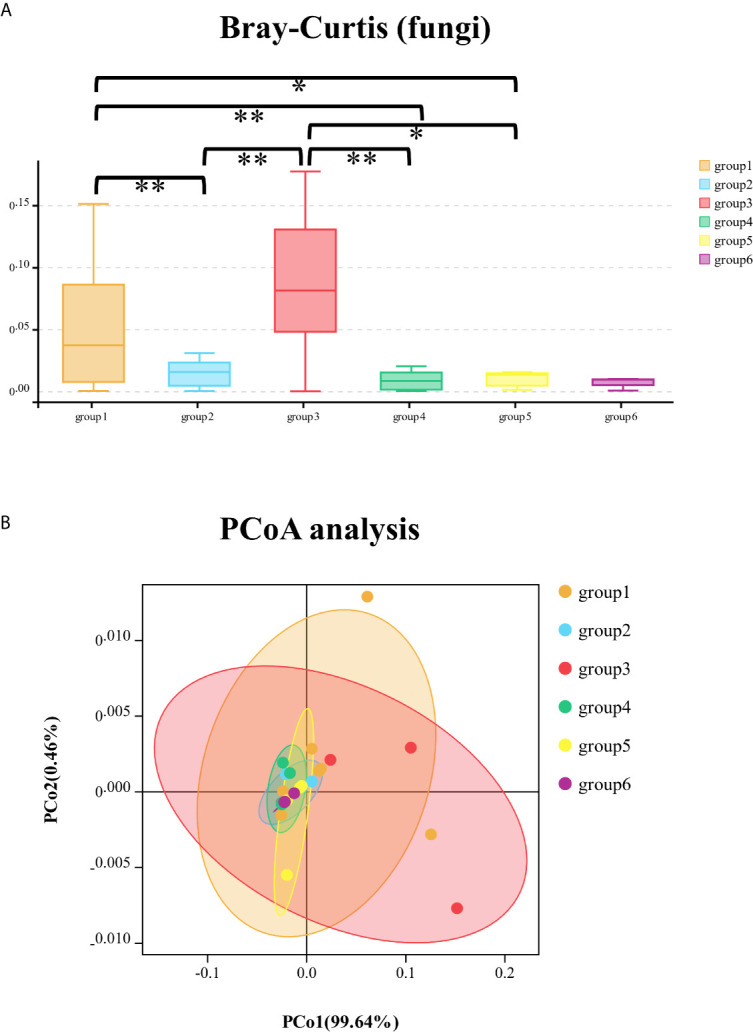
The comparison of Bray–Curtis distance of fungal community among six groups at phylum level **(A)**; principal co-ordinates analysis (PCoA) of fungal community among the six groups of samples **(B)**. *significant at *p* < 0.05, **significant at *p* < 0.01.

The fungal community of six groups had significant differences in Chao1 (Kruskal–Wallis test, *p* = 0.001) and ACE (Kruskal–Wallis test, *p* = 0.007) indices with Alpha-diversity analysis and were significantly different with the calculation of Bray–Curtis at phylum level (Kruskal–Wallis test, *p* < 0.001). The comparison of Bray–Curtis distance of fungal community among six groups at phylum level and PCoA analysis was illustrated (**Figure 3**). The genera of *Meyerozyma*, *Penicillium*, *Leohumicola*, and specie of *Candida_tropicalis* were different (*p* < 0.05) among the six groups.

### Microbiota of Biofilm Samples of DUWL Before and After Dental Practices

**Figure 4:**
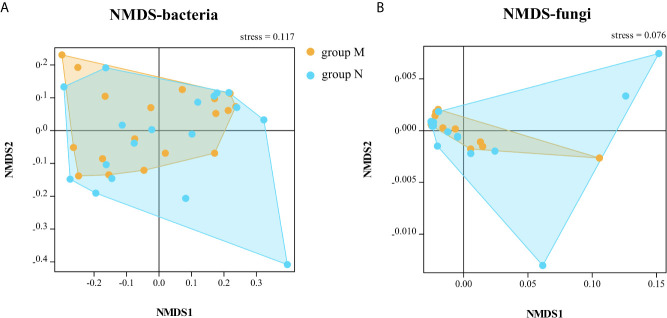
The non-metric multi-dimensional scaling (NMDS) analysis based on Bray–Curtis of bacterial (**A**, at family level) and fungal (**B**, at phylum level) communities before and after dental practice.

The bacterial community had no significant change in richness and evenness after one day’s dental practice according to analysis of all samples as a whole. The alpha and beta diversity analysis showed no significant difference between samples of DUWL before and after dental practices, except for under Bray–Curtis calculation at family level (Wilcoxon test, *p* = 0.04). There are some distinctions between two time points (before/after dental practice): Nitrospirae (0.006 *vs* 0.03%, *p* = 0.02) at phylum level; Phycisphaerae (0.07 *vs* 0.17%, *p* = 0.04) at class level; Actinomycetales (0.18 *vs* 0.02%, *p* = 0.01), and Pseudonocardiales (0.01 *vs* 0.08%, *p* = 0.04) at the order level; *Actinomyces* (0.16 *vs* 0.02%, *p* = 0.02), and *Sediminibacterium* (0.03 *vs* 0.11%, *p* = 0.04) at the genus level. Most bacteria increased, and only *Actinomyces* decreased after one day’s dental practices. The alpha diversity analysis of fungal community showed no significant difference between samples of DUWL before and after dental practices. However, the Bray–Curtis calculation at phylum (Wilcoxon test, *p* < 0.001) and species (Wilcoxon test, *p* = 0.009) levels showed significant differences. After one day’s work, Hymenochaetales (relative abundance 0.20 *vs <*0.01%, *p* = 0.04) and Tremellales (0.70 *vs <*0.01%, *p* = 0.03) nearly disappeared at the order level; Debaryomycetaceae (2.84 *vs* 0.41%, *p* = 0.04) decreased and Plectosphaerellaceae (0.03 *vs* 0.15%, *p* = 0.04) increased at family level; *Meyerozyma* (2.82 *vs* 0.41%, *p* = 0.03) decreased, and *Exophiala* (0.01 *vs* 0.07%, *p* < 0.01), *Cadophora* (<0.01 *vs* 0.49%, *p* = 0.01), *Verticillium* (<0.01 *vs* 0.15%, *p* < 0.01) increased at genus level. The NMDS analysis based on Bray–Curtis of bacterial (at family level) and fungal (at phylum level) communities before and after dental practices were illustrated (**Figure 4**).

### Environmental Factors Associated With DUWL Microbiota by Pearson Correlation Analysis

**Figure 5:**
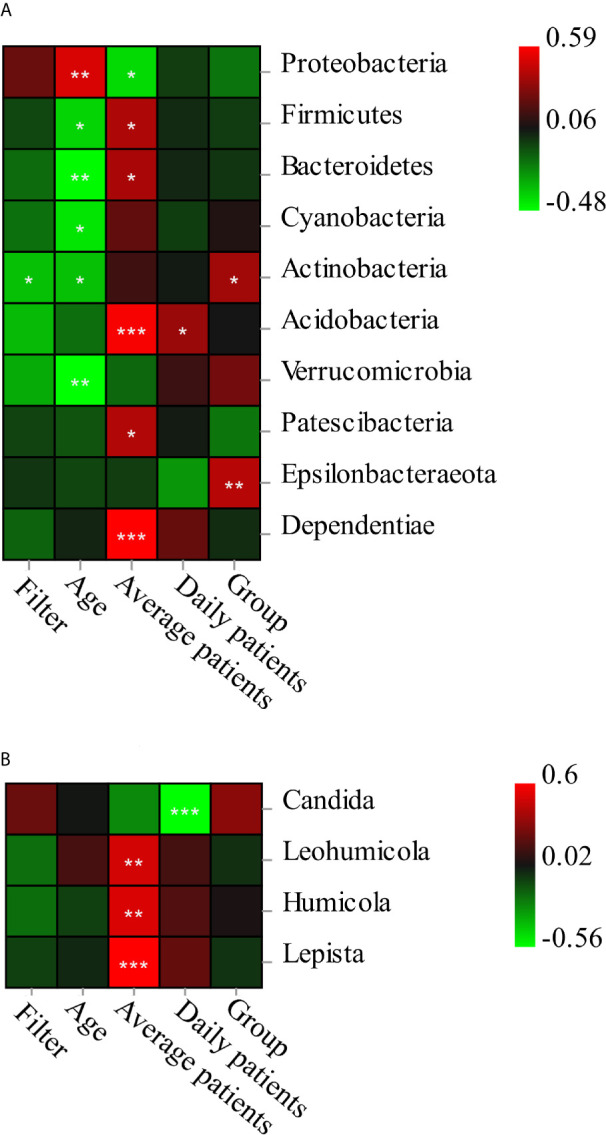
The Pearson correlation analysis of bacterial (**A** at phylum level)/fungi) (**B** at genus level) composition and five environmental factors. The interpretations of variables: filter-the application time of filter unit; age, age of dental unit; average patients, average patients per month; Daily patients-number of patients on the sampling day; Group-specialty of dental treatment. *significant at *p* < 0.05, **significant at *p* < 0.01, ***significant at *p* < 0.001.

Pearson correlation analysis revealed the microbiota composition was associated with environmental factors including the age of DCU (variable-Age), time of filter unit application (variable-Filter), average patients per month (variable-Average patients), group of specialty (variable-Group), and the number of patients on the sampling day (variable-Daily patients) **(** **Figure 5** **)**. As for the specific species, Acidobacteria and Dependentiae were positively related with the average patients per month (*p* < 0.001); Proteobacteria was positively related with age (*p* < 0.01) and negatively related with average patients per month (*p* < 0.05). Most genera of fungi were related with average patients per month including *Lepista* (*p* < 0.001), *Humicola* (*p* < 0.01), *Leohumicola* (*p* < 0.01), while *Candida* was negatively related with the number of daily patients (*p* < 0.001).

Variance partitioning analysis (VPA) showed that age (explanatory value = 19.64%) and average patients (explanatory value = 11.84%) contributed to phylum composition of bacteria, as well as class composition. Age (6.64%), filter unit (6.11%), and average patients (0.74%) accounted for order composition, as well as family composition; age (7.13%) and filter unit (5.69%) contributed to genus composition, while daily patients (0.62%) and filter unit (0.42%) contributed to the daily bacteria species variation. For fungal community of samples, group of specialty (3.36%) accounted for phylum composition; daily patients (13.60%), group of specialty (7.94%), and average patients (1.85%) accounted for class composition, as well as order composition; daily patients (19.18%), group of specialty (6.61%), average patients (2.58%), and filter unit (2.03%) accounted for species composition as well as genus and family composition. The age of dental unit and overall number of patients had the strongest impact on the overall bacterial composition, and the effect of daily dental practices (number of patients and specialty) on the fungi composition was the greatest.

## Discussion

### Microbial Communities in Biofilm of DUWL and Pathogens Related to Infection

This study revealed the DUWLs are heavily colonized by bacterial and fungal communities and investigated the associations with dental specialty and daily dental practices. We found that the most abundant phyla in biofilm samples were Proteobacteria (proportion of total sequences: 65.74–95.98%), Firmicutes (0.97–7.72%), and Bacteroidetes (0.73–5.40%), which was slightly different from previous studies which showed the pattern of Proteobacteria (32.78%), Tenericutes (31.76%) and Firmicutes (8.91%) (Zhang et al., 2018), or Proteobacteria and Actinobacteria as the two major phyla (Costa et al., 2015). The dominant fungi in this study were Ascomycota (93.9–99.3%) at the phylum level and Saccharomycetes, Eurotiomycetes, and Sordariomycetes at the class level. It was similar with another study which showed that Ascomycota and Basidiomycota at phylum level and the Saccharomycetes at class level were dominant in the core fungal microbiome (Costa et al., 2016b). The dominant fungi *Aspergillus*, *Candida*, and *Purpureocillium* in this study were also found in other research studies (Szymańska, 2005; Kadaifciler et al., 2013). These distinctions may be due to the different sample types (*i.e.*, biofilm and water samples) and methods (*i.e.*, sequencing directly or after cultivation). Previous study samples of DUWL included the supplying water, output water of dental instruments which were delivered through the narrow waterline tubes. Biofilm with proliferating of microcolonies formed and grew readily on the inner surface of DUWL (Porteous and Partida, 2009), showing different colonizers in early and late stages (Tall et al., 1995). It could be a reservoir of microorganisms and detach to the water continuously (Donnell et al., 2011), as well as protect the microorganisms from disinfectant by providing a suitable matrix of glycoproteins and polysaccharides (Walker et al., 2003). The relative abundance of Proteobacteria was obviously higher in the biofilm sample than in the water sample, possibly owing to the release of microbiota from biofilm to flowing water (Paramashivaiah et al., 2016), thus biofilm could better represent the true condition of DUWL rather than water sample and should be emphasized. In view of the variation of supplying water from different DCUs, the comparison of biofilm and water samples from the same DCU was needed to verify the distinction. There was a study (Szymańska, 2006) that found that the dominant fungal species were different among reservoirs water (*Candida curvata* and *Candida albicans*), handpieces water (*Candida albicans* and *Aspergillus glaucus*), and biofilm (*Aspergillus glaucus* and *Candida albicans*). Among the dominant bacteria, Proteobacteria was ubiquitous and physiologically versatile in drinking water (Vaz-Moreira et al., 2017) and even on earth (Zhou et al., 2020), which was related to bronchiectasis (Guan et al., 2018) and dysbiosis in gut microbiota (Shin et al., 2015). Heavier bacterial and fungal contamination in endodontics specialty was also observed in this study, which was consistent with a previous report (Zhang et al., 2018). The genus only detected in group of Endodontics*-Gardnerella* is atypical representative of oral cavity microflora, which has been detected both in vagina and oral cavity in people with bacterial vaginosis (Petrushanko et al., 2014). Except for *Gardnerella*, the relative abundance of *Sphingobium* was also higher in endodontics than in the other specialties, which may raise the risk in hospital tap water (Vaz-Moreira et al., 2011; Narciso-da-Rocha et al., 2014).

It is known that DUWLs are favorable environments for pathogen colonization. Among the six most abundant genera of opportunistic pathogens (*i.e.*, *Acinetobacter*, *Pseudomonas*, *Enterobacter*, *Aspergillus*, *Candida*, and *Penicillium*), high frequencies of bacterial respiratory pathogens, especially *Acinetobacter* spp. and *Pseudomonas* spp. were detected in oral cavity of patients (Zuanazzi et al., 2010). There are several reports about these pathogens with related diseases, such as *Pseudomonas aeruginosa* with lung infection in patients suffering from cystic fibrosis (Schick and Kassen, 2018), or with brain abscess (Pereira et al., 2017). Some opportunistic fungal pathogens (*e.g.*: *Penicillium*, *Candida*) isolated and identified in water samples from air-water syringes and high-speed drills may also lead to respiratory diseases such as allergic rhinitis (Kadaifciler et al., 2013). Other potentially pathogenic genera reported in a previous study (Costa et al., 2015) was also detected from our biofilm samples, including *Legionella*, *Mycobacterium, Propionibacterium*, *Stenotrophomonas*, *Flavobacterium*, *Streptococcus*, and *Escherichia-Shigella*. There are also numerous diseases probably associated with these pathogens, such as Legionellosis acquired through *Legionella* from a dental unit (Schönning et al., 2017); *Mycobacterium tuberculosis* and *Pseudoramibacter alactolyticus* coinfection in central nervous system after dental extraction (Liao et al., 2019); postoperative and device-related infections of the bones and joints, mouth, eye, and brain caused by *Propionibacterium* (Perry and Lambert, 2011). Apart from the pathogens, increased endotoxins of bacteria have been found in aerosols from DUWL with substantial biofilm growth, which may lead to inflammation of the airways (Szymanska and Sitkowska, 2013). Fungal pathogens can decrease host fitness by reducing survival and impacting host reproduction (Feistel et al., 2019). Compared with other similar research studies (Walker and Bradshaw, 2000; Walker et al., 2004) the rarely detected opportunistic pathogen species in this study, such as *Acinetobacter_lwoffii*, *Actinomyces_gerencseriae, Gardnerella_vaginalis, Staphylococcus_aureus, Streptococcus_pneumoniae, Candida_albicans, Exophiala_dermatitidis, Fusarium_solani, Stachybotrys_chartarum* might continue to remind us of the possible secondary infections. These results emphasized the potential adverse effects from DUWL despite the low species richness of these pathogens and the importance of regular control of microbiome contamination in DUWL.

### Environmental Factors Associated With Biofilm Accumulation

Among the possible environmental factors in this study, both age of DCU and daily dental treatment play a key role in biofilm accumulation. There was a study which showed that oral *streptococci* detection in water samples was not affected by handpiece age or dental treatment type, but was associated with dental unit age (Petti et al., 2013). Based on the high-throughput sequencing data of bacteria and fungi with environmental factors of this study, both specialty and daily dental practices would affect the microbiota composition; it may be due to the different uses of handpiece for different dental care, which was also associated with average of patients, for example, tooth cleaning and scaling in periodontics, tooth preparation and conditioning of prosthesis in prosthodontics (without too much detritus). However, it was less commonly used for orthodontics except for some polishing. The oral surgery specialty may probably use handpiece for splitting tooth with aerosol mixed with blood. It is shown that the pathogens of biofilms were also associated with dental specialty in view of a similar study showing that *Flavobacterium* was only detected in samples from the departments of endodontics and *streptococcus* was only found in samples from the department of periodontics (Zhang et al., 2018). *Candida albicans* is the most commonly isolated species from infected root canals (Sunde et al., 2002; Waltimo et al., 2003), which may affect the mycobiology of DUWL engaged in endodontics or pediatrics. The daily dental practice has certain effects on the microbiome of biofilm in DUWL; however, it must be taken into account that the two time points of sampling in one day may be not sufficient to screen biofilm accumulation; further longitudinal studies were essential to evaluate the dynamic accumulation.

Describing the problem of DUWL biofilm could emphasize on the contamination and biofilm control for dental chair unit manufacturers (Coleman et al., 2007). Despite disinfecting treatment and flushing process, microbial contamination remained relevant. Dental unit management is often missed or not correctly applied by stakeholders, with an underestimation of the real risk of infection for patients and operators (Lizzadro et al., 2019). Apart from the conventional measures of biofilm control (Donnell et al., 2011), there are some special advice in regard to manufacture, disinfectants and practice strategy. Rechargeable N-halamine-based antimicrobial functionality onto the inner surfaces of DUWL tubing (Luo et al., 2011), nanosilver disinfectant (Gitipour et al., 2017) could be taken into consideration. The hydrogen peroxide disinfection system was also proved in eradicating biofilm from DUWL and in controlling the bacterial count in water against several bacterial species (Orru et al., 2010). Some tested disinfectants active against sessile microorganisms were recommended to be used in a prophylactic rather than curative way (Costa et al., 2016a), and continuous disinfection was better than the intermittent treatment of DUWL (Laura et al., 2014). These supported that the filter unit used in the DCU of this study was an influencing factor on microbiota composition. Interestingly, the nature of DCU (associated with age and overall number of patients) and daily dental practices (associated with daily patients and dental specialty) were related with bacterial and fungal composition, respectively. For the dental staff, protective measures were also emphasized to avoid infections, especially in departments with large number of patients.

Compared with the method of sequencing after cultivation, we overcame the difficulties of insufficient quantity of microorganisms and explored a novel method of biofilm sample collection that could be generalized. Though high-throughput sequencing technology could afford huge amount of biological information, there are still some unclassified and unknown microorganisms with unclear risk. For the small sample size of some specialties of DUWL and insufficient days of observation, we should be cautious to conclude the distinctions of microbiota between specialties of DUWL. On the other hand, it should be noted that some non-living microorganisms may also be counted for sequencing with this method, and the infection risk may be overestimated. The results remind staff that they themselves and the patients may be exposed to many pathogens by inhalation or exposure during dental care and develop associated infections. This work was assumed to afford abundant information for biofilm control, and panic anxiety about the exposure to traces opportunity pathogens was unnecessary.

## Conclusion

To our knowledge, for the first time, biofilm in the DUWL engaged in different specialties was investigated by high-throughput sequencing.

- Both bacterial and fungal communities with heterogeneous and complex ecosystem of DUWL biofilm were revealed and were more abundant than in water samples with culturing method.
- The biofilm microbiome may be influenced by the characteristics of dental specialty and routine work to some extent. The age of DCU and overall number of patients had the strongest impact on the overall bacteria composition, and the effect of daily dental practices (associated with number of patients and dental specialty) on the fungi composition was the greatest.
- Considerable kinds of human opportunistic pathogens including both bacteria and fungi were detected in the DUWL biofilms, which suggested that dental staff and patients were at risk of potential infection despite the low species richness of these pathogens, and protective measures cannot be ignored.
- The novel method of biofilm collection from the plastic tube concatenated to dental instruments was feasible and can be generalized.
- This study demonstrated the necessity and importance of DUWL biofilm control and gave a clue for specific disinfection strategy.

## Data Availability Statement

The datasets presented in this study can be found in online repositories. The names of the repository/repositories and accession number(s) can be found below: https://www.ncbi.nlm.nih.gov/, PRJNA664509.

## Author Contributions

YH and CF contributed to conception and design of the study. CF, HG, and JY organized the data curation and validation. CF and YH performed the statistical analysis. HZ supervised the project. CF wrote the first draft of the manuscript. HG, LL, HZ, and JY wrote sections of the manuscript. All authors contributed to the article and approved the submitted version.

## Funding

This work was supported by the Youth Program of National Natural Science Foundation of China (No. 81700983).

## Conflict of Interest

The authors declare that the research was conducted in the absence of any commercial or financial relationships that could be construed as a potential conflict of interest.
